# Statistical aspects of discerning indel-type structural variation via DNA sequence alignment

**DOI:** 10.1186/1471-2164-10-359

**Published:** 2009-08-05

**Authors:** Michael C Wendl, Richard K Wilson

**Affiliations:** 1 The Genome Center and Department of Genetics, Washington University, St Louis MO 63108, USA

## Abstract

**Background:**

Structural variations in the form of DNA insertions and deletions are an important aspect of human genetics and especially relevant to medical disorders. Investigations have shown that such events can be detected via tell-tale discrepancies in the aligned lengths of paired-end DNA sequencing reads. Quantitative aspects underlying this method remain poorly understood, despite its importance and conceptual simplicity. We report the statistical theory characterizing the length-discrepancy scheme for Gaussian libraries, including coverage-related effects that preceding models are unable to account for.

**Results:**

Deletion and insertion statistics both depend heavily on physical coverage, but otherwise differ dramatically, refuting a commonly held doctrine of symmetry. Specifically, coverage restrictions render insertions much more difficult to capture. Increased read length has the counterintuitive effect of worsening insertion detection characteristics of short inserts. Variance in library insert length is also a critical factor here and should be minimized to the greatest degree possible. Conversely, no significant improvement would be realized in lowering fosmid variances beyond current levels. Detection power is examined under a straightforward alternative hypothesis and found to be generally acceptable. We also consider the proposition of characterizing variation over the entire spectrum of variant sizes under constant risk of false-positive errors. At 1% risk, many designs will leave a significant gap in the 100 to 200 bp neighborhood, requiring unacceptably high redundancies to compensate. We show that a few modifications largely close this gap and we give a few examples of feasible spectrum-covering designs.

**Conclusion:**

The theory resolves several outstanding issues and furnishes a general methodology for designing future projects from the standpoint of a spectrum-wide constant risk.

## Background

The relevance of genomic structural variation (SV) to human medical disorders is well-known [1,2] and efforts are starting to focus more systematically on characterizing SV and its implications [3]. Recent advances in technology [4], combined with the availability of the human genome sequence [5], are now opening dramatic new avenues of SV research [6-12]. These developments collectively point to the pending feasibility of investigating SV over its entire size spectrum. The most comprehensive projects will locate and identify variants, sequence them, and finally establish their statistical characteristics within a population [9].

**Figure 1:**
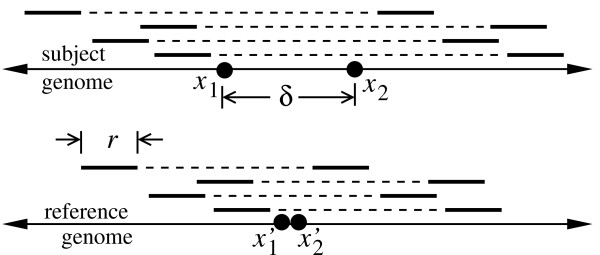
**Diagram of an insertion SV (ISV)**. Sequence inserts derived from the subject genome have a known average length, but appear to be shorter than average when their end-read pairs are aligned to a reference genome. This implies that segment , having length *δ *= |*x*_2 _- *x*_1_|, is inserted in the subject genome. Deletion SV (DSV) is the complement of this phenomenon and can be visualized by swapping the "subject" and "reference" labels in the diagram.

Broadly speaking, SV encompasses translocations, inversions, and copy number variations and other types of inserted and deleted sequences (indels). Here, we focus on the last category, which is believed to occur the most frequently [6]. Historically, cytogenetic techniques were used to examine instances of SV that were sufficiently coarse so as to be visible under a microscope [13]. Array technologies were later used heavily, but these platforms were still not able to reliably capture alterations well below 40 kb [14]. More recently, Volik *et al*. [15] proposed a procedure based on paired-end sequences that can detect much smaller variants, depending upon the type of sequence insert one employs. The scheme is remarkably straightforward in concept, relying on the fact that if the subject genome contains an insertion or deletion structural variant (ISV or DSV, respectively), the length statistics of any paired-ends aligned to a reference genome will differ from those of the progenitor library. Specifically, inserts would appear to be longer and shorter on average, respectively, for DSV and ISV (Fig. 1). The method basically furnishes a metaphorical caliper for observing the tell-tale length discrepancies that characterize SV.

Although investigators are actively pursuing this technique [6-12], it is still somewhat new and its conceptual simplicity actually belies a number of latent complications. Alignment tasks are not trivial [16,17], nor are accurate descriptions of a host of statistical issues. For instance, breakpoint localization has only been examined under the idealization of constant insert lengths [18]. Gaussian length distributions provide a much better empirical fit. Indeed, projects routinely invoke precisely this assumption, subsequently exploiting elementary Gaussian thresholds to define their SV detection framework. For example, a common rule has been to declare SV if the aligned average length differs from the library average by at least 3 standard deviations [6,10,11,15]. This threshold implies a confidence interval of slightly better than 99%, or equivalently, the chance of committing a false positive classification error of *α *< 1%. Other procedures call for considering inserts more than 2 deviations from the average [17].

In actuality, the statistical aspects of this problem are rather more complicated than what the above practices would suggest. One of the outstanding issues is coverage, which current theory ignores entirely [6,10]. Traditional fixed-length processing models [19-22] are not particularly useful here because the local covering dynamics will depend upon the variation of insert lengths in the library. While the role of variability has actually been recognized for some time [20,23], it has not been formally investigated much beyond the elementary uniform distribution model [24,25]. Consequently, there is little understanding of how the main statistical classifiers, *α *and *β *(Table 1), are affected by Gaussian variance through the mechanism of coverage. A subtext to this point is that the statistics of ISV versus DSV are not symmetric, as is commonly assumed [6,10,11,15]. Finally, it appears that there have been no comprehensive studies related to the statistical power of the method or to how the spectrum of SV sizes can be effectively managed in a project.

**Table 1:** Notation for Structural Variation (SV) statistics

Variable	Meaning
Probabilistic Descriptors of SV Detection	

*α*	probability of false-positive errors
*β*	probability of false-negative errors

Genomic and Project Parameters	

*λ*	average insert length in a Gaussian library
*σ*	standard deviation in a Gaussian library
*δ*	length of an instance of structural variation
*r*	(constant) sequencing read length
*τ*	difference threshold specified for power analysis
*N*	number of inserts processed
*G*	haploid subject genome length
*m*	minimum admissible insert size (Eq. 5)
*ρ*	haploid physical coverage (≡ *N λ*/*G*)

Labels Defining Types of SV	

ISV	insertion SV
DSV	deletion SV
*H*_*t*_	heterozygous SV
*H*_*m*_	homozygous SV

Functions and Random Variables	

erf	Gaussian error function (see e.g. Ref. [28])
exp	exponential function
	random number of inserts spanning an SV site
*ℒ*	random length of an individual insert
*ℳ*	random mean length of inserts spanning SV site

All of these issues have important implications for the broader enterprise of SV research, from project planning and optimization to defining detection rules within SV algorithms. Here, we report the mathematical analyses that lead to a general *a priori *statistical characterization of ISV and DSV when using the length-discrepancy technique in conjunction with Gaussian libraries. We describe several novel aspects of SV detection revealed by this theory and comment on their implications for pending SV projects.

## Results

Alignments to the reference genome for which one or more inserts seem either abnormally long or short *may *represent instances of SV (Fig. 1). In the theoretically ideal case of identical insert sizes, the task of SV identification is elementary. A single spanning insert is sufficient for an un-ambiguous assignment, i.e. *α *= 0, and the detection power is simply the local coverage probability, 1 - *e*^-*ρ*^, where *ρ *is the redundancy [19]. The actual problem is rendered more difficult by the natural length variability present in all libraries; It tends to obscure the ability to differentiate true SV-related length differences from those arising merely from the population sampling effect [26]. Although methodology-related artifacts can also arise, e.g. anomalous mappings of chimeric or extremely small reads, we do not explicitly investigate these second-order effects.

Consider a library in which the insert length ℒ is Gaussian (normally) distributed [26,27] as(1)

where *λ *and *σ *are the average and standard deviation of length, respectively, and exp is the exponential function. End-pair alignment of randomly selected inserts yields a sample whose members span a candidate SV region having a magnitude at least *δ *= |*x*_2 _- *x*_1_| (Fig. 1). The associated length statistics that we wish to describe are functions of several variables (Table 1) and are governed by what we call the sampling and covering problems. Their solutions can subsequently be combined in a number of ways to represent the various possibilities of considering SV.

### The Sampling Problem

Let random variables  and ℳ represent the number of randomly selected inserts that span a candidate SV site and the mean length of these inserts, respectively. The *sampling problem *requires the determination of confidence intervals on ℳ with respect to the size of SV being examined. That is, if the probability of ℳ being within *λ *± *δ *by virtue of random sampling effects is very high, then any actual average falling outside these limits would be significant. Such an observation would strongly imply an instance of SV of size at least *δ*.

#### Theorem 1 (Sampling Probability)

Define the null hypothesis, *h*_0_, as the absence of SV of size *δ*. Given *k *inserts spanning , the two-tailed test for *h*_0 _is(2)

where erf is the Gaussian error function [28]. Eq. 2 gives the sampling probability that a perceived event of ISV ∪ DSV will actually fall within the established confidence interval due to random sampling effects. Conversely, the sampling probability of either type of SV event considered alone, i.e. as a one-tailed test is(3)

where these represent ISV and DSV, respectively.

### The Covering Problem

The *covering problem *addresses the question of how many inserts span  and is complicated by several factors. First, there are non-trivial difficulties in the alignment process [16], necessitating several simplifying, but reasonable assumptions. Given fixed sequencing read lengths of *r *(Fig. 1), we do not consider cases in which a read, rather than its progenitor insert, intersects the boundaries of . Such instances would lead to read-splitting in DSV and read-truncation in ISV. This scenario is actually the basis of an altogether different kind of detection technique [29], which we do not discuss here. So-called "singleton reads" can arise if the missing mate originated entirely within an ISV, and we briefly discuss this possibility further below.

The second complication is that the statistics of ISV and DSV are not actually symmetric, as is commonly presumed [6,10,11,15]. For example, in the strictest case, we do not admit singleton reads in ISV, meaning the placement constraints are much more restrictive than for DSV. Lastly, the Gaussian model itself introduces certain mathematical difficulties.

#### Lemma 2 (Bernoulli Covering Probability)

Consider the event *S *in which a single insert of length *l *(Eq. 1) spans a site of SV. Let *H*_*m *_and *H*_*t *_represent the status of the SV as homozygous and heterozygous, respectively. If the genomic source has a haploid length *G*, the Bernoulli probabilities of *S *are(4)

Where(5)

is the minimum admissible insert size and *l ≥ m *(Eq. 11).

If singleton reads are allowed for ISV, then *m *would actually be smaller than the expression given in Eq. 5, however, there is no reliable basis for measuring insert length in such cases, even for perfect, un-ambiguous alignments [16]. Consequently, there is an irreconcilable mismatch between the numbers of covering and sampling inserts. In the interest of being able to make direct comparisons to the established symmetric models using a two-tailed test [6,10,11,15], one could simply, though somewhat erroneously, take *m *= 2*r *for these cases.

#### Theorem 3 (Coverage Distribution)

 is Poisson distributed [26,27] with a rate(6)

where(7)

is the general rate expression and *N *is the number of inserts that have been processed.

### Statistical Models of SV

The classification problem for SV is characterized by the probabilities of incorrectly calling out an instance of SV (a false positive) or overlooking a true instance of SV (a false negative). Such scenarios are described by the traditional inference probabilities *α *and *β*, respectively [26], which can be constructed from the above components. We discuss some of the various combinations here, again taking the null hypothesis, *h*_0_, as the absence of SV of size at least *δ*.

#### Theorem 4 (General Characterization of False-Positives)

Let *C *be the event that the sample average length falls within some specified confidence interval, then(8)

is the probability that differences between the sample and library average lengths are attributable to the sampling effect [26]. Instances falling outside the interval imply rejecting *h*_0 _and these are significant at a level of *α *= 1 - *P*(*C*). This statement immediately implies the following corollaries.

#### Corollary 5 (Standard Model)

The standard model recognizes the inherent asymmetry between ISV and DSV, employing separate one-tailed tests for each. Specifically *C *represents intervals *λ *- *δ *≤ ℳ and ℳ ≤ *λ *+ *δ *for ISV and DSV, respectively, where the variable *m *embedded in these equations assumes appropriate values, as given by Eq. 5. Homozygous and heterozygous configurations are differentiated according to Eq. 6.

#### Corollary 6 (Symmetric Model)

The symmetric model disregards the asymmetry between ISV and DSV and implements classification according to the two-tailed test, where *C *is the interval *λ *- *δ *≤ ℳ ≤ *λ *+ *δ *and where *m *= 2*r *for both ISV and DSV. Homozygous and heterozygous configurations are again differentiated according to Eq. 6. This model can be regarded as a direct extension of previous work [6,10,11,15], though it harbors the singleton anomaly discussed above.

#### Theorem 7 (Elementary Characterization of False-Negatives)

Let *D *be the event that an instance of SV is *provisionally *detected (i.e. not overlooked), as defined under the following conditions: It is spanned by at least one insert and the length of at least one of those spanners is different from the library mean by a specified threshold amount *τ*. We have(9)

where *t *= *τ*/(*σ*). Consequently, the detection power *P*(*D*), sometimes written more traditionally as 1 - *β*, is given by Eq. 8, where *C *is replaced by *D*.

## Discussion

Here, we examine some of the consequent properties of the so-called length-discrepancy method that will have implications for future projects. This discussion is framed in terms of several insert types that are in either experimental or common use (Table 2). We concentrate largely on the most conservative event from the standpoint of detection: heterozygous insertion.

**Table 2:** Representative insert types for discovery over the SV spectrum

insert type	*λ *(kb)	COV (%)	*r *(bp)
Illumina GA short^*a*^	0.25	12	50
Illumina GA intermediate^*b*^	3	12	50
454^*c*^	3.2	25	250
Illumina GA long^*d*^	10	12	50
fosmid^*e*^	39.9	7	600
BAC^*f*^	136.4	21	600

^*a *^representative of < 1 kb inserts, see e.g. refs. [4,29]

^*b *^insert length representative of extended chemistry protocols for 2–5 kb inserts, see e.g. ref. [33]

^*c *^library parameters reported in ref. [8]

^*d *^experimental, not currently in routine use

^*e *^library parameters reported in ref. [6]

^*f *^primary breast tumor library B421, see ref. [11]

### Overview of Trends for False-Positive Calls

**Figure 2:**
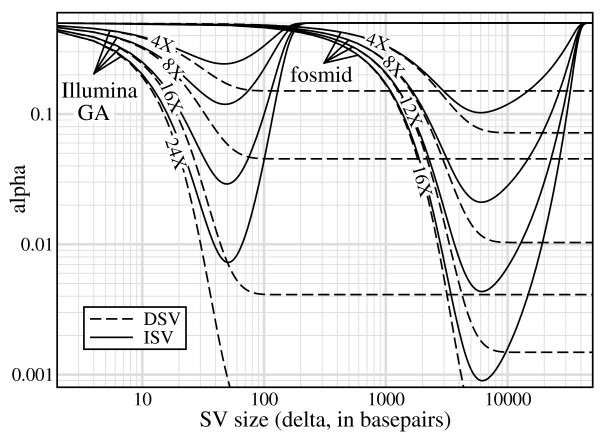
**Heterozygous ISV and DSV false-positive trends for 250 bp Illumina GA inserts and 40 kb fosmids (Table 2) for selected values of physical coverage (*ρ *= *N λ*/*G*)**.

Thus far, concerns have predominantly focused on the rate of false-positive SV declarations. The general methodology has been one of assuming symmetric behavior of ISV and DSV and subsequently employing elementary Gaussian thresholds, usually ± 3 *σ*, to control false-positive errors [6,9-11,15]. For example, Tuzun *et al*. [6] aimed to identify SV of size *δ *≥ 8 kb using fosmids. Fig. 2 revisits this aspect of the problem with respect to heterozygous SV for the edge cases of Illumina Genome Analyzer (GA) short inserts and large-insert fosmids (Table 2). Here, we demonstrate that false-positive behavior is more complex than what simple Gaussian thresholds are able to capture.

The asymmetry between ISV and DSV is quite clear. Statistical significance of DSV detection is basically governed by a single minimum size, *δ*_*min*_, for each specific case. For example, the significance level *α *= 1% for fosmids is realized for roughly all *δ *≥ 8 kb at *ρ *= 8× physical coverage. While matching the *δ*_*min *_= 8 kb calculated by Tuzun *et al*. [6], this datum is purely a coincidence. Asymptotic *α *is actually highly dependent upon the physical coverage, as shown in the figure, while *δ*_*min *_is only weakly dependent upon *ρ*.

The situation for ISV is quite different in that there is not only a *δ*_*min*_, but also a maximum size, *δ*_*max*_. The latter arises because placement constraints for fixed *λ *increase with ISV size. Physical coverage is again tightly coupled with these limits. Increased *ρ *not only renders the better (lower) values of *α *more accessible, but also widens the range of acceptable *δ*. For example, heterozygous ISV using fosmids does not register at the 1% significance level even at 8× physical coverage. However, at 12×, events are significant roughly in the range 3,700 ≤ *δ *≤ 12,700 and at 16× in 2,800 ≤ *δ *≤ 19,200. The upper limit on *δ *increases somewhat faster with redundancy than does the lower limit for ISV.

While trends are similar for Illumina GA short inserts, there are notable differences with respect to physical coverage. In particular, ISV at 1% significance does not become feasible until roughly 24×, and even then its range is quite small. The main parametric differences from fosmids are that the variance is higher here, 12% coefficient of variation (COV) vs. 7% reported in ref. [6], and that read length is a much greater fraction of insert length, i.e. *r*/*λ *= 20% here vs. about 1.5% for fosmids. Increased variance obviously degrades the statistics, but ironically, so do "better" read lengths. The latter phenomenon arises because of the requirement that reads lie outside the insertion, which implies fewer placement possibilities for covering (Eqs. 4 and 5). These observations indicate that short-read data will generally have to be generated at much deeper redundancies than large-insert clones (discussed in more detail below) and also raise the issue of optimal read lengths. However, the latter depend on all the methods one might use to find variation, so it cannot be settled on the basis of the length-discrepancy approach alone.

All ISV curves approach the asymptote *α *= 50% as *δ *increases as a consequence of vanishing covering probabilities. Inferences in these regions are no better than a coin flip. Notice that the fosmid curves also approach the 50% asymptote as *δ *→ 0. The underlying factor here is a vanishing precision, not unlike that experienced when evaluating the small difference between two increasingly large numbers.

### Remarks on Detection Power

The concept of detection power is never entirely precise because it requires adoption of *ad hoc *alternatives to the null hypothesis [30]. Ours rests on a simple, but intuitive presumption: detection is only possible if at least one insert spans the SV site, and if at least one of these spanners has a length sufficiently different from the library average, as specified by *τ*. For instance, for ISV, at least one covering insert must be shorter than *λ *- *τ*, meaning its aligned length will be less than *λ *- *τ *- *δ*.

**Figure 3:**
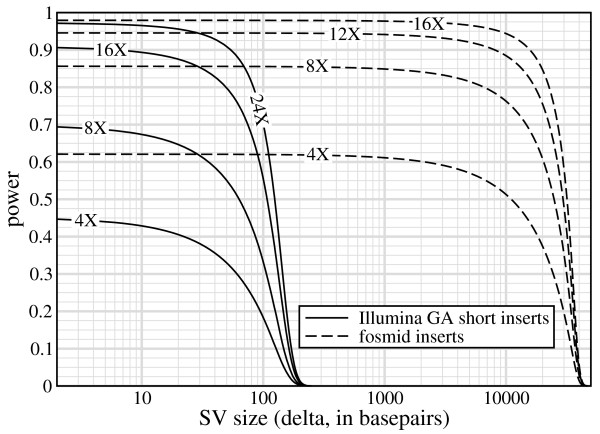
**Heterozygous ISV power for short Illumina GA inserts and fosmids (Table 2) at *τ *= 0**.

Thresholds can be specified in numerous functional ways, each of which has certain implications for finding SV of different sizes. Fig. 3 examines the idealized scenario of *τ *= 0 for heterozygous ISV. Each curve is asymptotic for *δ *≪ *λ*, but rapidly decays as SV size approaches the insert size.

Asymptotics are readily shown as a special case of the model to be(10)

In fact, Eq. 10 also represents the (constant) power for DSV. Here again, we see that inserts having relatively long reads, i.e. larger r/*λ*, are penalized, but increased redundancy can compensate for the shortfall. For instance, asymptotic power for Illumina GA data could be made equal to that for fosmids if its redundancy were roughly 60% higher than fosmid redundancy. Note the trend for each insert's power curve to more visibly resemble a unit step function as redundancy is increased. In comparing *α *to power, it appears the latter is quite acceptable. For example, 16× fosmids are upper-bounded at roughly 20 kb for *α *= 1% significance (Fig. 2), for which the corresponding power is still about 85%. This is notable because the choice of *τ *= 0 is not especially sensitive for large insertions.

Defining rigorous detection rules is a challenging task for algorithm developers. Again using ISV as an example, the aligned length of the shortest covering insert, *l*_*s *_- *δ*, will be known, although *l*_*s *_and *δ *themselves generally are not. However, one can readily quantify power versus the aligned length of the *average *insert, for example using some *τ *= *δ*/*C*. This information might serve as a basis for constructing detection rules by correlating *a priori *known theoretical entities with other heuristic information.

### The Nature of Library Variance

**Figure 4:**
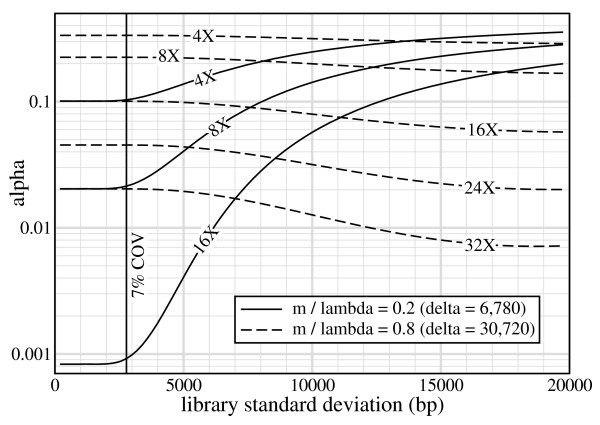
**Curves of *α *vs *σ *for heterozygous ISV using 40 kb fosmids on "small" (*m*/*λ *= 0.2) and "large" (*m*/*λ *= 0.8) insertions**. Vertical reference line shows the 7% COV threshold, characteristic of the library in ref. [6].

Library variance is conventionally thought of as something that should be minimized to the greatest extent possible in order to improve SV detection [9]. This view actually comes with some caveats, as illustrated by Fig. 4 for heterozygous ISV detection using 40 kb fosmids. Fosmid libraries can routinely achieve COV of around 7% because of packaging constraints inherent to the vector [6]. Yet, *α *is largely constant for COV ≤ 7% over a wide range of redundancies, implying that special efforts aimed at further reducing fosmid library variance would be unwarranted. While some sensitivity is actually realized for very small ISV, i.e. less than 10% of insert size, the limit on precision mentioned above renders these instances irrelevant.

Another curious phenomenon associated with ISV lurks in Eq. 7. Its exponential and error function terms have leading coefficients *σ *and *λ *- *m*, respectively. The second term represents the familiar "Lander-Waterman" type of covering mechanism which should ideally provide the bulk contribution, but its potency drops considerably for larger *δ *via *m *(Eq. 5). This reflects the simple fact that the *average *insert will not cover very well under these circumstances. Performance can be recovered in a seemingly counter-intuitive way by *increasing *the library variance, which implies there are more longer-than-average inserts in the library. Fig. 4 confirms this effect, although it is evidently not substantial enough at reasonable redundancies.

**Figure 5:**
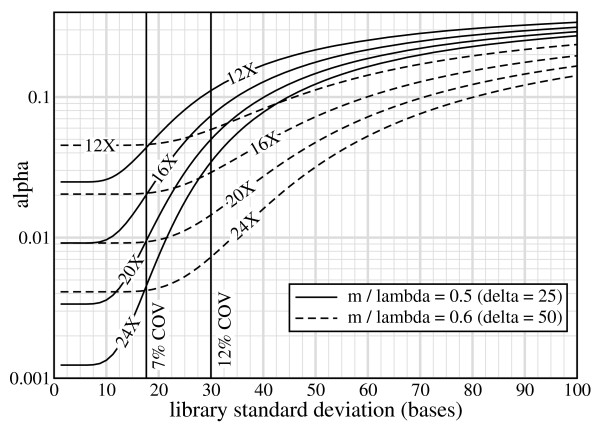
**Curves of *α *vs *σ *for Illumina GA small inserts at two intermediate sizes of heterozygous ISV: *δ *= 25 bp (*m*/*λ *= 0.5) and *δ *= 50 bp (*m*/*λ *= 0.6)**. The 12% COV characteristic of this library is shown by a vertical line. A second line at 7% COV is given as a reference to the fosmid library in ref. [6].

The situation is appreciably different for Illumina GA inserts, where *α *rapidly becomes responsive to library variance over slight changes in *m*/*λ *(Fig. 5). It is mildly sensitive at *m*/*λ *= 0.6 (50 bp ISV), meaning that there would be some level of improvement if library standard deviation could be reduced. However, for a small decrease to *m*/*λ *= 0.5 (25 bp ISV), the situation worsens in two ways. Not only does *α *become appreciably more sensitive, but attempts to compensate with increased redundancy are less effective. For example, at 12% COV we could reduce *α *by 68% (from 11% to 3.5%) by doubling *ρ *from 12× to 24×. If Illumina GA libraries were improved to fosmid-level 7% COV, we would instead see *α *reduced by 90%, from 4.3% to 0.44%. Yet, the curves show that still more resolution could be wrung out, all the way down to about 4% COV (*σ *= 10 bp). This overall behavior is again a consequence of the relatively long read lengths, which drive down *δ *for a given *m*/*λ *ratio. Although these observations suggest investing more effort into reducing *σ *for Illumina GA libraries, the balance against economic considerations has not been conclusively established.

### The SV Spectrum Problem in Project Design

SV projects are becoming both more routine and more focused on characterizing the entire SV size spectrum. One of the more pressing design questions with respect to length-discrepancy analysis is how to a *priori *specify the combination of insert types and corresponding redundancies that will best characterize the relevant fraction of the spectrum [12], roughly 10 ≤ *δ *≤ 20,000 bp. (Other methods are better-suited outside these ranges, for example some arrayCGH platforms can detect variants down to the 20–30 kb range [14].) Investigators will typically want to capture all types of SV, implying a project's design will be governed by the most difficult type: heterozygous insertions. We examine the problem primarily on this basis, but emphasize that the findings presented below must be interpreted within the larger context of a sequencing project whose considerations are not limited strictly to SV, much less a single method of detecting it.

**Figure 6:**
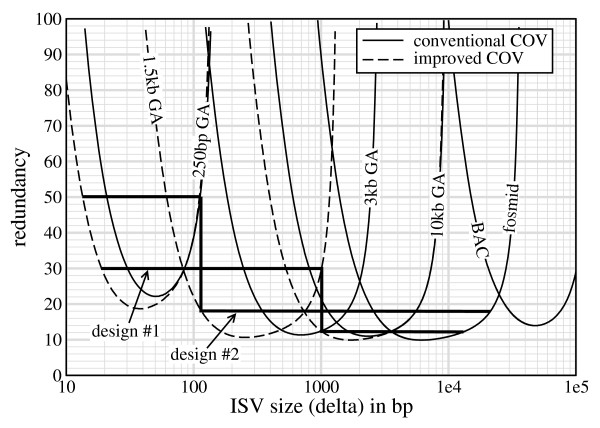
**Spectral curves for heterozygous ISV at a threshold of *α *= 1% for both conventional insert types (Table 2) and hypothetical Illumina GA inserts having one-third lower (improved) COV values**. Bold lines represent two feasible designs that leave no spectral gaps at *α *= 1% using the improved GA libraries.

Fig. 6 addresses the design issue from the standpoint of the "spectral chart", which is readily plotted from the theory. In particular, the solid curves represent the loci of points at which the desired *α*, in this case 1% [6,10,11,15], is realized for the conventional inserts listed in Table 2. (We omit the pyrosequencing-454 insert, whose relatively large COV renders it less suitable for SV applications compared to the comparable Illumina GA library [8].) The dashed curves denote improved performance of hypothetical Illumina GA libraries whose COVs are one-third lower than conventional values.

Medium and large Illumina GA inserts, combined with fosmids, readily handle SV above about 300 bp using assorted physical coverages in the 15× – 20× range. Although the small insert library does cover much of the neighborhood on the lower end, there is a conspicuous gap between 100 and 200 bp, precisely where many variants could be expected [10]. Redundancies for both the small and medium insert libraries would have to increase to roughly 90× to close this gap, an obviously undesirable requirement. Yet, matters would improve considerably with a few design adjustments.

Let us assume hypothetical Illumina GA libraries whose COVs are reduced by one-third of their conventional values. Also, replace the 3 kb Illumina GA library with a 1.5 kb GA library. These two modifications largely erase the gap, i.e. curves for adjacent libraries now intersect at roughly 30×. Switching to the 1.5 kb library is primarily responsible for this closure, although reduced COVs further improve spectral coverage on the lower ends of the respective libraries. This effect is especially relevant to extending detection range for the smallest SVs.

Spectral charts are useful for designing projects according to the requirement that no gaps remain in the SV detection spectrum for a desired *α*. Fig. 6 shows two such designs. The first proposes roughly *ρ *= 30× for 250 bp and 1.5 kb GA libraries and *ρ *= 12× for 10 kb GA and 40 kb fosmid libraries, netting SV within approximately 20 ≤ *δ *≤ 13000. The second prescribes 50× for the 250 bp GA library and 18× for the remaining 3 libraries, widening the range to roughly 13 ≤ *δ *≤ 21000. Although many other designs are clearly possible, these illustrate some of the interesting trade-offs faced by the investigator. For example, maximally extending the range raises the possibility of including additional insert types, e.g. a BAG library. Costs are also vastly different over the various insert types, with small GA being the cheapest, larger GA being more costly because of library inefficiencies, and fosmids being the most expensive according to direct library and sequencing costs. However, these issues are tempered by the fact that long inserts efficiently leverage *sequence *redundancy, 2 *ρ r*/*λ*. For example, 20× fosmid physical coverage translates to only about 0.5× sequence redundancy [10]. The second design in Fig. 6 is probably superior to the first from this standpoint.

## Conclusion

Our theory describes SV statistics in more general terms than currently available, though it still depends upon a number of idealizations for the sake of tractability. Specifically, the covering process is taken to be independently and identically distributed. We also presume genome size is known a *priori*, which may not be the case for tumors. Finally, we do not account for mapping or sequencing errors, library complexity, the ability of algorithms to distinguish between the reads covering both alleles of a heterozygous SV site [17], instances of singleton, split, and truncated reads, etc. The strength of any predictions should be taken with these limitations in mind.

Design optimization is clearly important and we have only considered it from the rudimentary perspective of eliminating gaps in the SV size spectrum. Indeed, there are numerous possible designs for any spectral chart, as well as numerous charts that could be plotted by varying the types and numbers of libraries, values for *α*, etc. It would be quite useful to optimize on the basis of total project cost, but variation in library production and sequencing costs among different insert types, lab environments, production methods, bio-economical feasibility of minimizing COV for inserts, etc. places this goal beyond the present scope. Results shown here suggest an intermediate number of libraries, i.e. three to five, will typically give the best results. Too few will incur extremely large per-library redundancies because of spectral gaps and the asymptotic nature of spectral coverage (Fig. 6), while too many will result in unacceptably high production costs. Very roughly speaking, each insert type should probably be responsible for up to an order of magnitude of SV size.

Aside from the project design aspects, our theory should also be useful for SV algorithms. Many algorithms still follow the symmetric assumption where DSV and ISV are considered to be simple opposites [17], though they clearly are not. Bayesian classifiers might invoke Thm. 3 in calculating prior probabilities. The theory might also be useful in helping to pick optimal threshold values, for example with respect to detection power, as mentioned above. Finally, the overall design space is enormous and certainly worth further exploration, especially in conjunction with better bio-economical information on the feasibility of reducing COV for Illumina GA libraries. Future projects could carry out such investigations for neighborhoods of interest in a fairly mechanical fashion.

## Methods

This section furnishes proofs of the above theorems and describes the analytical and numerical methods used. These mathematical esoterica can be skipped by the uninterested reader.

### Proof of Theorem 1

If insert lengths are Gaussian distributed with standard deviation *σ *(Eq. 1), then the mean length ℳ of random samples of size *k *(the aligned inserts) will be Gaussian with standard deviation *σ*_*S *_= *σ*/[26]. The probability of the confidence interval is defined as [27]

where (*a'*, *b'*) = (*λ *- *δ*, *λ *+ *δ*) for Eq. 2, but change to *a' *→ - ∞ or *b' *→ ∞, as appropriate, in Eq. 3. By making a change of variables, *x *= (*l' *- *λ*)/(*σ*_*S*_), the theorem of Integration by Substitution [31] can be invoked to obtain

where erf is the Gaussian error function [28] and where limits have been transformed appropriately. For example, *λ *- *δ *is changed to -*δ*/(*σ*_*S*_) and *λ *+ *δ *to *δ*/(*σ*_*S*_). All intervals follow directly by substituting *σ*_*S *_= *σ*/ and applying the identities erf (-*x*) = -erf (*x*) and erf (∞) = 1 [28], the latter where necessary.

### Proof of Lemma 2

Lemma 2 is proved by straightforward enumeration. First, there are roughly 2*G *possible placements of any insert, since the haploid genome and its constituent chromosomes are very large. For homozygous SV, the apparent number of placements is then only *G*. For DSV, we disqualify cases where either read of an insert intersects an SV site, whereby the spanning event is realized only if the interior region between the paired reads contains , which is a point in this case. There are *l *- 2*r *placements satisfying this condition, implying spanning probabilities of (*l *- 2*r*)/*G *and (*l *-2*r*)/(2*G*) for *H*_*m *_and *H*_*t*_, respectively. For ISV, the breakpoints are separated, and the variant exists as segment  (Fig. 1). The number of successful covering placements is *l *- 2*r *- *δ *+ 1, which is well-approximated as *l *- 2*r *- *δ*, given that *l *≫ 1. Spanning probabilities for *H*_*m *_and *H*_*t *_follow by appropriate division. The fact that 2*r *and 2*r *+ *δ *are the minimum admissible insert lengths for DSV and ISV, respectively, is a direct consequence of both the mathematical fact that probability values cannot be negative and the physical observation that these are the minimum values at which an insert could span the respective types of SV.

### Proof of Theorem 3

Based on a Bernoulli probability *P*(*S*) for each insert (Lemma 2), the covering process for a site of possible SV is clearly binomial, i.e. an individual insert either covers, or does not cover the site. For SV projects, the number of inserts processed, *N*, and *P*(*S*) are necessarily large and small relative to unity, respectively. The Poisson distribution for *P *( = *k*) then follows directly from the standard binomial limiting argument [27], where *μ *= *N*·*P*(*S*) is the Poisson rate. We will demonstrate only the homozygous configuration, since the heterozygous case follows along exactly the same arguments.

The prior Bernoulli probability of event *S*, whereby an insert covers a possible site of SV, is conditioned upon its length. Consequently, it can be expressed according to the Law of Total Probability [27] as(11)

where the summation appears by virtue of the events being mutually exclusive. Note that *P *(ℒ = *l*) and *P*(*S*|ℒ = *l *∩ *H*_*m*_) have already been established by Eq. 1 and Lemma 2, respectively. The latter also establishes *m *as the lower bound, while the upper bound is a consequence of the observation that inserts will not exceed the subject haploid DNA length. Biological constraints actually restrict inserts to much smaller sizes. However, it will be clear momentarily that the actual value of the upper bound is not particularly important because the associated functions rapidly converge to their respective limits.

The form of *P*(*S*) is further developed by first transforming to an integral representation. Substituting *P *(ℒ = *l*) and *P*(*S*|ℒ = *l *∩ *H*_*m*_) and moving constants outside the integral, we find

where the error of transformation can be shown by the Euler-MacLaurin Theorem [28] to be

Because *λ *≫ 1, even for inserts considered to be fairly short, and likewise *σ *≫ 1, the magnitude of *ϵ *will be acceptably small for cases of practical interest. Making a change of variables similar to that shown in the proof of Theorem 1 the integral evaluates to

Given that *G *is extremely large (order 10^9 ^for the human genome), the second exponential term vanishes, while the first error function term is asymptotically equivalent to unity. (Since the nature of both kinds of terms is to converge rather rapidly, these limits would still be realized for substantially lower values.) Applying the identity erf (-*x*) = -erf (*x*) [28] to the second error function term gives the final form of *P*(*S*), from which *μ *= *N*·*P*(*S*) in Eqs. 6 and 7 immediately follow.

### Proof of Theorem 4

The event *C *whereby the average length of the sampled inserts falls within a certain confidence interval is conditioned upon how many inserts actually comprise the sample, whereby from the Law of Total Probability [27] we find

from which Eq. 8 follows directly.

### Proof of Theorem 7

Using the same integral methods shown in the proof for Thm. 1, the probability of selecting any single insert *i *whose length *L*_*i *_is within some range of *τ *is

where *t *= *τ*/(*σ*). Because inserts are presumed independent of one another, the probability that *k *inserts picked from the library are all within the specified range is *P*_1 _∩ *P*_2 _∩ ⋯ ∩ *P*_*k *_= , whereby the probability that at least one is not in range is the complement of this expression. The overall probability of detection under the above model is conditioned upon *k *inserts spanning the SV site, whereby application of the concept of Total Probability (similar to what is shown in proof of Thm. 4) yields *P*(*D*).

### Numerical Methods

The statistical properties of SV depend upon numerical evaluation of *P *( = *k*), whose floating-point numerator and denominator will both tend to overflow as *k *grows large. In these cases, we evaluate *P *( = *k*) in logarithmic form. Using Stirling's Series for the factorial term [28]

we find that the expression can be written

for large *k*. Given the definition of redundancy in Table 1, it is numerically more expedient to replace *N*/*G *with *ρ*/*λ *in Eq. 7. Also, it will often be impractical to sum all *N *+ 1 terms, e.g. in Eq. 8, so we use a simple convergence rule that halts the computation when the percentage change due to the current term falls below a small number, typically 10^-8^.

Accurate numerical methods exist for evaluating the Gaussian error function [32], and such are available in the functional compendium of most of the common programming languages, including C/C++, Fortran, Mathematica, and Perl.

## Abbreviations

SV: structural variation; COV: coefficient of variation.

## Authors' contributions

MCW conceived and constructed the mathematical theory and wrote the paper. Both authors approved the final manuscript.
